# Pharyngoplasty With Suture of Mucosal Flap Complementary Coverage for Treating Pharyngeal Stenosis

**DOI:** 10.1002/ccr3.70156

**Published:** 2025-02-04

**Authors:** Zhiyan Lu, Yimiao Wang, Dan Li, Siyi Chen, Shuaichi Ma, Peijie He

**Affiliations:** ^1^ Department of Otorhinolaryngology, Eye and ENT Hospital, ENT Institute Fudan University Shanghai China

**Keywords:** caustic ingestion, CO_2_ laser, endoscopic surgery, pharyngeal stenosis

## Abstract

Exploring a new method of pharyngoplasty surgery, which uses CO_2_ laser to dislocate the mucosa and then covers the wound with mucosal flap complementary sutures, successfully preventing postoperative pharyngeal stenosis.

## Graphical abstract

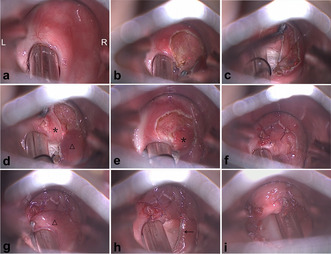

**Summary**

- A 9‐year‐old male patient suffered pharyngeal stenosis as a result of caustic ingestion, which manifested as dyspnea and dysarthria.
- The patient underwent pharyngoplasty with mucosal dislocation incision and complementary suture.
- During a follow‐up period of 6 and 10 months, the patient's dyspnea resolved entirely, and his phonation showed significant improvement.

## 1 Introduction

Pharyngeal stenosis can be congenital or acquired. At present, congenital pharyngeal stenosis has been reported more frequently [1], while acquired pharyngeal stenosis, especially those caused by corrosive injury, has been reported less frequently. There are even fewer cases in children. This article presents a case report of a pediatric patient who developed pharyngeal stenosis as a result of caustic ingestion. Additionally, we propose an innovative surgical technique for the management of pharyngeal stenosis, which has the potential to prevent adhesion formation and recurrence of stenosis.

## 2 Case History/Examination

The patient is a 9‐year‐old male. 6 years ago, he swallowed some detergent, a strong alkaline substance by accident. Despite promptly expectorating the substance, he presented with lip edema and oral burning. Upon immediate hospital admission, gastroscopy revealed esophageal injury. Antibiotics and glucocorticoid were given intravenously. Due to the patient's inability to consume food orally, a nasogastric tube was inserted to ensure adequate nutritional support. After 7 days of treatment, the patient exhibited no symptoms, including hoarseness, dysphagia, or dyspnea, and was subsequently discharged after the removal of the nasogastric tube. Pharyngeal stenosis was not found on examination at that time. However, 1 month after injury, the patient manifested respiratory symptoms such as dyspnea and cough. Although tracheoscopy evaluation revealed pronounced pharyngeal stenosis, surgical treatment was deemed unnecessary due to the patient's young age and the insufficiency of symptom severity to pose a significant impact on quality of life.

**FIGURE 1:**
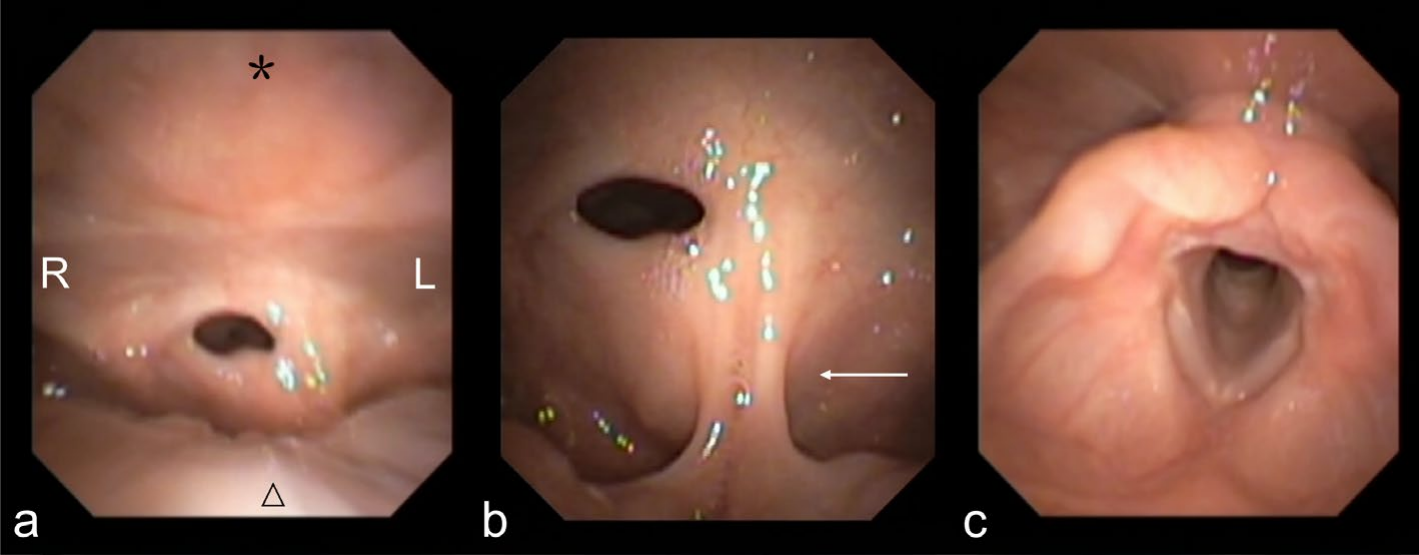
Preoperative laryngoscopy (a) The epiglottis stump is adhered to the posterior pharyngeal wall (asterisk). Triangle indicates root of tongue. (b) A magnified view of the pharyngeal cavity is presented, with arrows indicating the middle glossoepiglottic fold. (c) Normal laryngeal structure is observable through the small hole.

Before current admission, the patient's symptoms of dyspnea, wheezing, and dysarthria worsened without obvious inducement, while symptoms such as hoarseness and dysphagia were absent. At admission, fibrolaryngoscopy showed a defect in the upper epiglottis, with adhesion of the epiglottic stump to the posterior pharyngeal wall, and a small hole remaining on the right side (Figure 1a,b). The aryepiglottic folds, false and true vocal folds were smooth (Figure 1c).

## 3 Treatment

**FIGURE 2:**
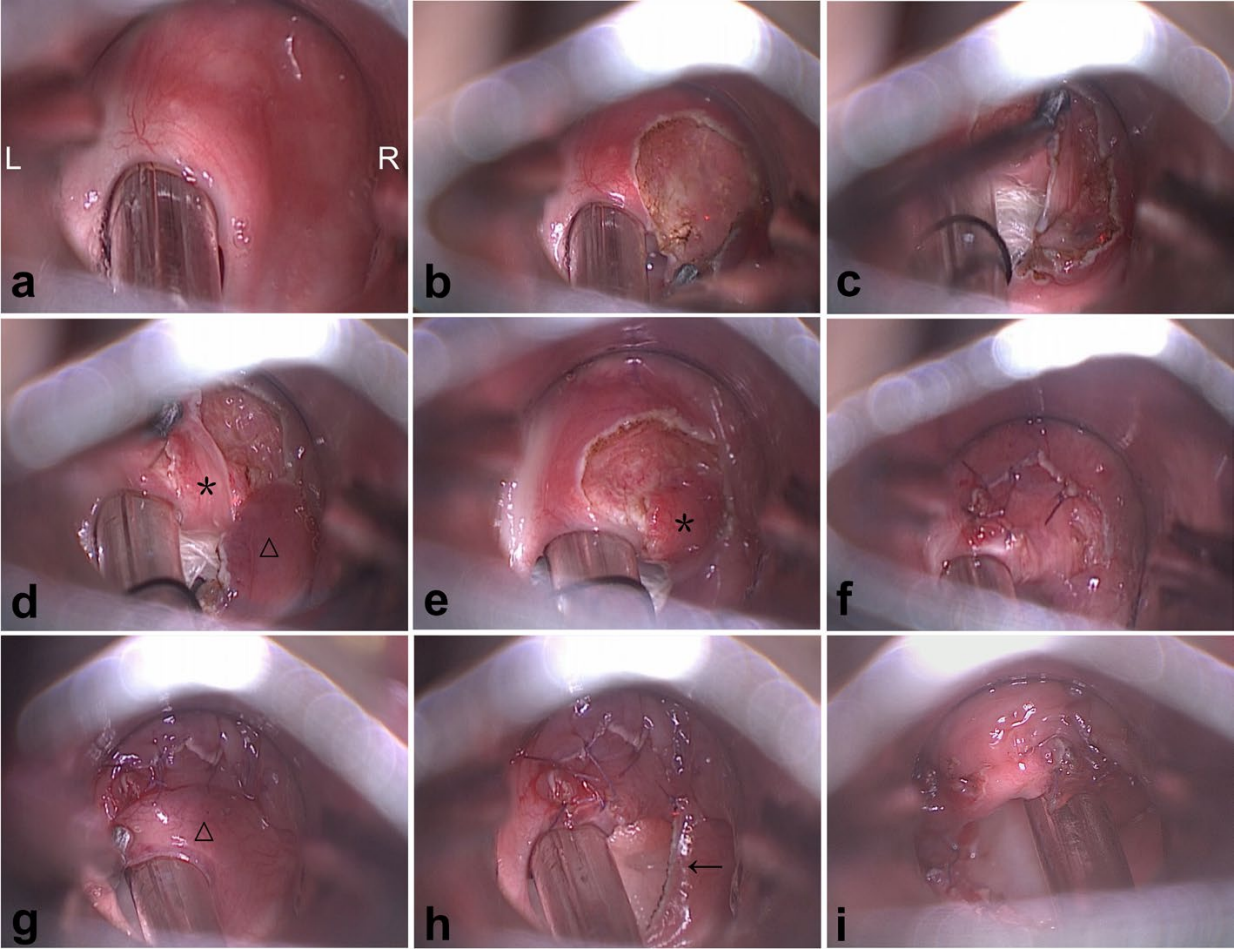
Surgical procedure (a) Preoperative view. The top of the image is the epiglottic stump. (b) The superior mucosal flap is removed. (c–e) The mucosa is further incised and detached from the posterior pharyngeal wall while the inferior mucosal flap (asterisk) is repositioned from facing the larynx to facing the tongue. The triangle indicates the excess mucosa. (f) The mucosal flap is sutured. (g) The triangle again indicates the excess mucosa. (h) The excess mucosa is excised. The incisal edge of the lateral pharyngeal wall (arrow) remains exposed and is yet to be folded down and sutured. (i) The postoperative view demonstrates a satisfactory shape of the epiglottis.

The patient was admitted to our hospital in November 2023. After discussion, our department decided to conduct adhesion incision and pharyngoplasty using CO_2_ laser 3 days after admission. After the laryngeal surface anesthesia with fiber bronchoscope, the patient was intubated awake with the light stick. Once general anesthesia was induced, the patient was positioned supine with the head back. Under microscopic guidance, a moist cotton pledget, approximately 6 mm in diameter, was placed around the anesthesia cannula to protect the anesthesia tube balloon. Before incision, the anesthesiologist was informed to switch the anesthetic gas to mixed gas. We created the two‐layer mucosal flaps by utilizing CO_2_ laser (2 W, continuous mode) (DEKA SmartXide2C60 M103F1). Specifically, the adherent pharyngeal mucosa was divided into a superior layer (the side facing the tongue) and an inferior layer (the side facing the larynx). The superior mucosa of the right side was excised first, followed by the incision of the inferior mucosa, which was then separated from the right lateral pharyngeal wall. The inferior mucosa was repositioned from an orientation facing the larynx to one facing the tongue using laryngeal forceps. To shape the right epiglottic mucosa, a 6–0 absorbable suture was used to suture the mucosal flap and the incisal margin of the lingual surface mucosa of the epiglottis together. The epiglottic mucosa was preserved and the excess mucosa was excised from the lateral pharyngeal wall. The mucosal flap on the lateral pharyngeal wall was sutured to the lateral edge of the mucosa to contour the hypopharynx (Figure 2). The same procedure was applied to the left side.

## 4 Outcome and Follow‐Up

**FIGURE 3:**
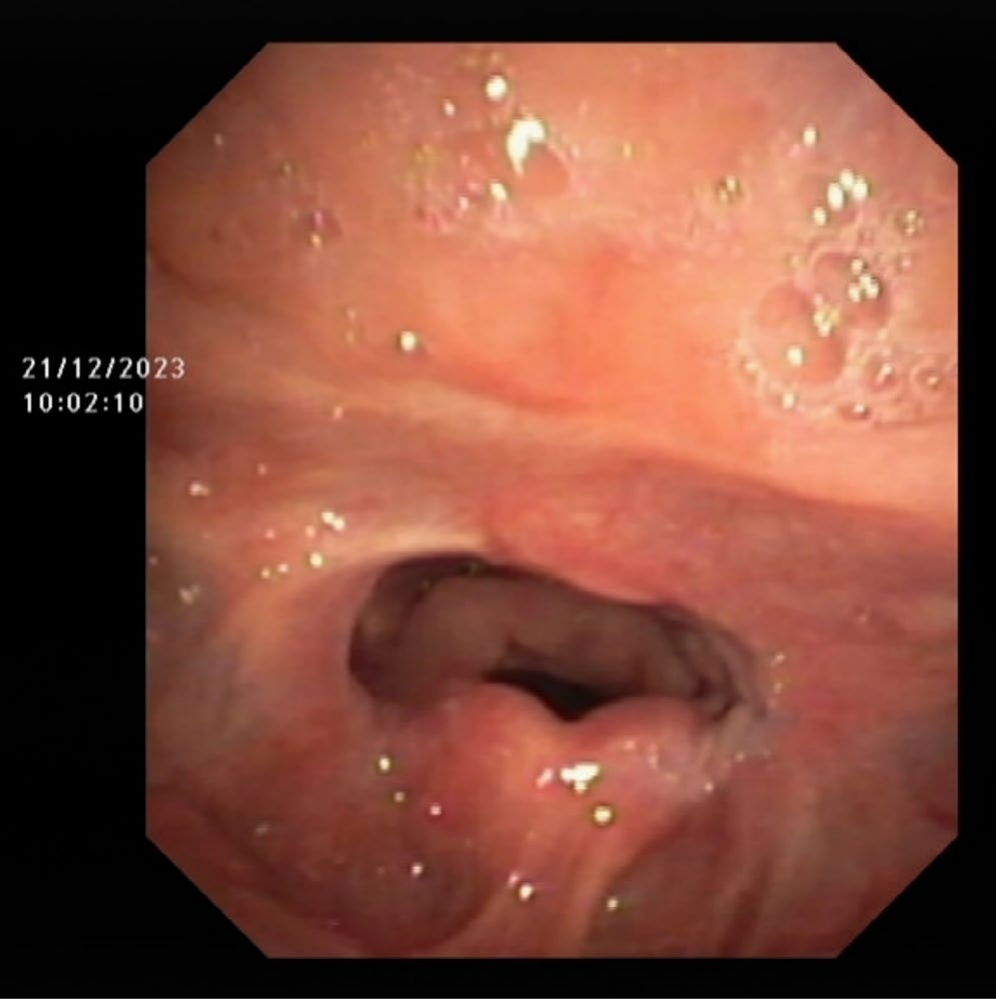
Endoscopy performed 21 days after surgery.

During the treatment, neither tracheotomy nor nasogastric tube insertion was performed. The patient was able to consume food orally without choking on the first day after surgery, and there was a marked improvement in the symptom of dyspnea. Laryngoscopy conducted 3 weeks post‐operatively showed that there was no recurrence of pharyngeal stenosis (Figure 3). During a follow‐up phone call 6 and 10 months after the surgery, the patient's parents reported the resolution of dyspnea and an improvement in dysarthria.

## 5 Discussion

Caustic ingestion of household cleaner by children aged 2–6 years accounted for 80% of cases of caustic injury. The severity of the injury depends on a number of factors, such as the chemical nature (acidic or basic), physical state, and quantity of the substance ingested. Acids induce coagulation necrosis, resulting in cicatrices, while alkalis cause liquefaction necrosis, leading to immediate and severe penetrating damage. As to physical form, the rapid flow of liquid substances not only damages the mouth and pharynx but also causes digestive tract burns [2, 3, 4].

Caustic ingestion can lead to a variety of complications, accompanied by diverse symptoms. Hoarseness, wheezing, aphonia, and dyspnea suggest laryngeal edema or epiglottis and laryngeal destruction. Due to the protective effect of the epiglottis, the vocal cords are generally not damaged, which explains why hoarseness is uncommon in these patients [5, 6]. Endoscopy in the acute stage of injury remains to be discussed and should be selected cautiously according to the actual situation of the patient, because the invasive maneuvers may aggravate the injury. Non‐contact examinations such as CT and even laryngeal ultrasonography [7] are optional means. Pharyngeal stenosis is a potential complication of caustic injury, typically manifesting 1–2 months after the initial injury. Therefore, the diagnosis and treatment also should be emphasized even after the acute phase.

Pharyngeal stenosis is a complication arising from ingestion of harmful substances, total laryngectomy, chemoradiation, or malignant tumors. The cases of pharyngeal stenosis surgery caused by caustic ingestion are relatively rare. Although uncommon, the diagnosis of this case as pharyngeal stenosis is explicit, and what matters is how to treat it and prevent it from recurring. Both Berlucchi [6] and Velasco [5] chose to remove the stenosis directly with CO_2_ laser and applying mitomycin C to prevent the stenosis. The patients had undergone tracheotomy and gastrostomy before surgery, and finally could successfully achieve extubation postoperatively. We sought improvement surgically upon this foundation.

Preventing fibrosis and recurrence postoperatively remains a significant challenge for surgeons. Regardless of whether a cold instrument or a CO_2_ laser is employed, simple excision of the tissue tends to elevate the risk of postoperative recurrence and scar formation. Many researchers have worked on this issue. The study of laryngeal stenosis has received more attention compared to pharyngeal stenosis. The implementation of a laryngeal stent and the grafting of intervening tissue have been demonstrated to enhance phonation [8, 9]. Yılmaz [10] first proposed the concept of butterfly mucosal flap. The sutured flaps covered the exposed surfaces, thus diminishing the risk of postoperative laryngeal web reformation. Both subjective and objective assessments of voice quality and ventilation demonstrated significant improvement with this technique. In opinion of the author, in addition to the application of chemical agents such as mitomycin and steroids, these methods primarily aim to minimize contact with surgical wounds to prevent recurrence. This technique adheres to the same underlying principle.

The excision and preservation of adhesive mucosa presented a significant concern in our study. In the surgical treatment of glottic web, the removal of excess mucosa remains a contentious issue. Schweinfurth [11] recommended removal of excess mucosa directly, while Yılmaz [10] suggested that the mucosal flap was insufficient to cover the entire exposed mucosal surface and thus should be preserved. However, this issue does not manifest in the region of hypopharyngeal stenosis due to the comparatively larger mucosal surface area relative to the larynx. But there is a fact that the flap shrinks during the developmental process. It should be noted that the flap should not be excessively small and that the tension should not be excessively high during the creation and suturing of the mucosal flap.

Coordination with the anesthesiologist is crucial, given that airway manipulation and stenosis may impede safe management under general anesthesia. At the beginning of the operation, the anesthesiologist should stop supplying pure oxygen and instead supply mixed air to prevent the airway from burning due to the laser.

Tracheotomy and insertion of nasogastric tube after pharyngoplasty are often difficult to avoid. In this case, the patient's parents exhibited significant resistance to both tracheotomy and nasogastric feeding. Finally, these interventions were not employed, and the patient achieved a satisfactory therapeutic outcome.

It is the first case in which CO_2_ ablation and mucosal flap suture have been applied conjunctively to the treatment of pharyngeal stenosis caused by caustic ingestion. The innovative approach demonstrated promising outcomes. By adhering to the principle of covering exposed mucosal wounds, this technique holds potential applicability to similar conditions, such as pharyngeal webs, but further research and clinical validation are necessary. Our research does have certain limitations, such as recent laryngoscope was unavailable due to the kid's intolerance, which may render our therapeutic outcome less convincing. But the clinical effect is lasting and stable. Parents were satisfied and subjectively perceived that the patient's condition had not deteriorated in last 10 months.

While this case is atypical, the occurrence of caustic ingestion and pharyngeal stenosis is relatively common. Our findings may have broader implications for similar cases.

## Author Contributions

**Zhiyan Lu:** conceptualization, formal analysis, investigation, methodology, writing – original draft. **Yimiao Wang:** investigation, validation. **Dan Li:** writing – review and editing. **Siyi Chen:** investigation. **Shuaichi Ma:** software. **Peijie He:** conceptualization, funding acquisition, resources, supervision, validation, writing – review and editing.

## Ethics Statement

The investigation has been approved by the Institutional Research Ethics Committee of EENT Hospital and that the investigators have obtained written informed consent from participant and guardians.

## Conflicts of Interest

The authors declare no conflicts of interest.

## Data Availability

The data that support the findings of this study are available from the corresponding author upon reasonable request.
